# Claudins play a role in normal and tumor cell motility

**DOI:** 10.1186/1471-2121-14-19

**Published:** 2013-03-23

**Authors:** Patricia G Webb, Monique A Spillman, Heidi K Baumgartner

**Affiliations:** 1 Department of Obstetrics and Gynecology, University of Colorado Anschutz Medical Campus, Reproductive Sciences Mail Stop 8613, Aurora, CO 80045, USA

**Keywords:** Claudin, Motility, Collagen, Mammary cells, Breast cancer, Ovarian cancer

## Abstract

**Background:**

Claudins are key integral proteins of the tight junction. Although they play an essential role in controlling paracellular diffusion in epithelia, increasing evidence supports a role for these proteins in non-barrier forming activities. To elucidate a potential function for claudins outside of their traditional role in tight junctions, subcellular localization of claudin-4 was determined in normal mammary epithelial cells as well as breast and ovarian cancer cell lines and the effects of a claudin mimic peptide on cell motility were determined.

**Results:**

Immunofluorescence revealed that claudin-4 was localized along cellular projections. Using a fluorescent peptide that mimics a conserved sequence in the second extracellular loop of a set of claudin subtypes, that includes claudin-4, exposure of this loop to the extracellular environment was confirmed in non-polarized cells. This peptide inhibited cell motility when normal mammary epithelial cells as well as breast and ovarian tumor cells were subjected to a wound healing assay. Knockdown of claudin-4 also inhibited cell motility and the mimic peptide had no effect on motility in the claudin-4 knockdown cells. This effect on motility was seen when cells were grown on collagen, but not when cells were grown on non-physiological cell adhesive or fibronectin.

**Conclusion:**

The second extracellular loop of claudins is able to interact with the extracellular environment to promote normal and tumor cell motility when it is not associated with tight junction structures.

## Background

Claudins are important transmembrane proteins in tight junctions. They are best known for their homo- and heterotypic interactions between adjacent epithelial cells that control the tissue-specific paracellular permeability properties of epithelia. Although the structure and functions of the 27 different subtypes [1] of claudin are not fully understood, the tight junction barrier properties of claudins have been well characterized. Immunohistochemistry and immunofluorescence analysis of claudin localization in normal tissue shows that some claudin subtypes are not restricted to the tight junction. Extra-junctional claudins have been traditionally attributed to storage and mobilization of claudins to and from the tight junction. For example, Shen [2] and colleagues have elegantly demonstrated through FRAP (recovery of fluorescence after photobleaching) analysis that the tight junction is constantly remodeling. However, Blackman et al. [3] have shown that claudin-7 is present in mammary epithelial cells at fair levels at all stages of development but never localizes to the tight junction. Instead, it is found in cytosolic vesicle-like structures near the basolateral membrane in these cells as well as cells in the pulmonary bronchus and the renal cortex. Basolateral localization of claudin-1, –3, –4, –7 and –8 has also been identified in intestinal epithelium by Chiba et al. [4] and Ding et al. [5] Ding and colleagues found that intestinal function was seriously compromised in claudin-7 knockout mice. This observation suggests that non-junctional stores of claudin may potentially be involved in activities of normal epithelia in addition to replenishing tight junction claudin.

A potential function for claudins not localized within tight junction structures can also be supported by the observation that during tumor progression, where disruption and loss of tight junction structures is a hallmark characteristic, certain claudin subtypes are distinctly expressed. Claudins -3, –4 and -7 are often present in breast [6-8], ovarian [9-11], and endometrial [12,13] tumors and are often expressed at elevated levels. Using serial analysis of gene expression (SAGE), Kominsky and coworkers have shown both that claudin-4 is expressed in the majority of breast tumors tested and that expression is increased 2–3 fold compared to normal breast tissue [14]. Furthermore, claudin-3 and -4 are not normally expressed in ovarian surface epithelium, but are highly expressed in 70% of ovarian tumors [10]. Although over expression of claudins is well documented in epithelial-derived cancer cells, the role of claudins in tumor promotion has received little attention.

Increasing evidence supports a role for claudin proteins in cell motility. Agarwal and colleagues observed an increase in cell motility when normal human ovarian surface epithelial cells (HOSE) were transfected with claudin-3 or claudin-4 compared to non-transfected HOSE cells, which do not express these claudins [15]. A similar phenomenon was observed in gastric adenocarcinoma cells, where forced over expression of claudin-6, –7, and -9 increased the motility of these cells compared to non-transfected gastric adenocarcinoma cells [16]. Alternatively, the loss of claudin-11 (also known as oligodendrocyte-specific protein) was shown to impair migration of primary oligodendrocytes [17] and loss of claudin-5 in the breast cancer cell line MDA-MB-231 cells inhibited cell motility [18]. The mechanisms by which claudins influence cell motility are not well understood. However, a few studies have found claudins within protein complexes that contain important motility molecules such as CD44v6/EpCAM/CO-092 in colorectal cancer cells [19] and N-WASP/ROCK in metastatic breast cancer cells [18]. Miyamori and colleagues [20] have also shown that claudin-1, –2, –3, and -5 have the potential to interact with the MT1-MMP metalloproteinase and that this interaction can enhance the processing and activation of MMP-2, an important metalloproteinase involved in promoting cell motility via degradation of the extracellular matrix.

To further clarify how claudins may function as both a tight junction protein as well as a potential motility molecule, we have focused our attention on the proposed structure of claudin. Claudins span the membrane four times, with cytosolic N- and C-terminal domains and two extracellular loops. This structure gives them the potential to mediate interactions between the intracellular and extracellular environment. The cytosolic C-terminal domain of claudins contains a PDZ-binding domain that is known to bind the cytoplasmic plaque proteins ZO-1, ZO-2, and ZO-3 [21] thus linking the tight junction to the cytoskeleton. Signaling molecules, such as MAGI, MUPP1 and Par-3, are also known to contain PDZ domains and although the PDZ binding protein partners of claudin have received little study, there is a strong potential for claudins to play an important role in cell signaling mechanisms. The extracellular loops of claudin are also key domains that are known to contribute to claudin function. These loops are known to interact with specific claudin subtypes on opposing epithelial cells as well as with claudin subtypes within the same plasma membrane [22-24]. Claudin extracellular loops have also been shown to interact with occludin [25], another integral protein of the tight junction. These claudin-claudin and claudin-occludin interactions are important for forming the protein strands of the tight junction. During pathological conditions, claudin extracellular loops are targeted by and interact with pathogens such as hepatitis C virus [26] and *Clostridium perfringens* enterotoxin [27]. The question remains whether or not the extracellular loops of claudins may normally interact with components of the extracellular milieu such as the extracellular matrix proteins, particularly since claudins have been found localized at or near basolateral membranes of normal epithelium.

In this study we investigated the potential function of claudin-4 in promoting cell motility, specifically testing the hypothesis that claudin-4 directs cell movement through extracellular loop interactions. With immunofluorescence, we found claudin-4 localized along cellular projections of both normal and tumor cells. Using a small peptide that mimics a conserved sequence in the second extracellular loop of subset of claudin subtypes, including claudin-4 [28], we were able to determine that the second extracellular loop of non-tight junctional claudins is exposed to the extracellular environment in non-polarized cells and that interruption of this loop’s normal interactions inhibits cell motility. The inhibition of cell motility is strongest with cells plated on collagen, suggesting a potential interaction of claudin with extracellular molecules to promote cell movement.

## Results

### Claudins are found in cellular projections

**Figure 1:**
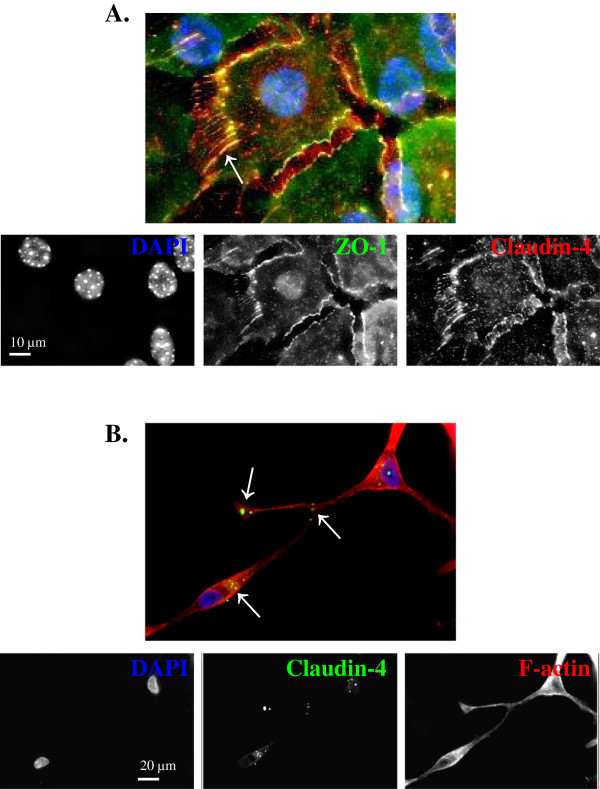
**Claudin-4 localization in normal and tumor cells. **Representative confocal microscopy images of fixed subconfluent primary mammary epithelial cells (**A**) and breast cancer 21PT cells (**B**) treated with antibodies directed to claudin-4 (**A**: red, **B**: green) and/or ZO-1 (**A**: green) and stained with DAPI (**A**&**B**: blue) and/or phalloidin (**B**: red). Claudin-4 localizes within distinct puncta in the cytosol near the nucleus as well as along cellular projections. Arrows point to claudin-4 puncta in cellular projections in both normal and tumor cells. Yellow color of the merged image (**A**) indicates overlay of the red claudin and the green ZO-1 immunofluorescence.

We first used immunostaining to localize claudin-4 in both normal mammary epithelial cells and breast tumor cells. Previously, we had demonstrated that localization of claudin-3 and claudin-4 is restricted to the tight junction in confluent monolayers of normal mouse mammary epithelium in culture, using the established cell line EpH4 [28] as well as primary mammary epithelium isolated from wild type FVB mice [29]. However, when we examined claudin-4 localization in these primary mammary epithelial cultures before they reached confluence, we found it within distinct puncta along thread-like projections between adjacent cells (Figure 1A). Claudin-4 co-localized with the tight junction protein ZO-1 at the cell boarders as well as in a few of the cell projections. This zipper-like appearance is similar to what is seen in early or primordial junction formation. To determine whether claudin could be found within cellular projections in cells that lack the ability to form tight junction structures, we examined localization of claudin-4 in breast cancer cells. We chose several breast cancer cell lines to investigate. A breast cancer progression series, which includes a cell line isolated from a primary breast tumor (21PT) and a line isolated from a metastatic lesion (21MT) from the same patient, was first examined. Claudin-4 appeared in distinct cytosolic puncta or vesicle-like structures, often found on one side of the cell or along cellular projections in non-confluent cultured cells. These puncta were common at sites where the projection touched another cell and at the end of the projections (Figure 1B). Unexpectedly, a similar pattern of claudin-4 localization was seen in both the primary and metastatic cells lines (data not shown).

**Figure 2:**
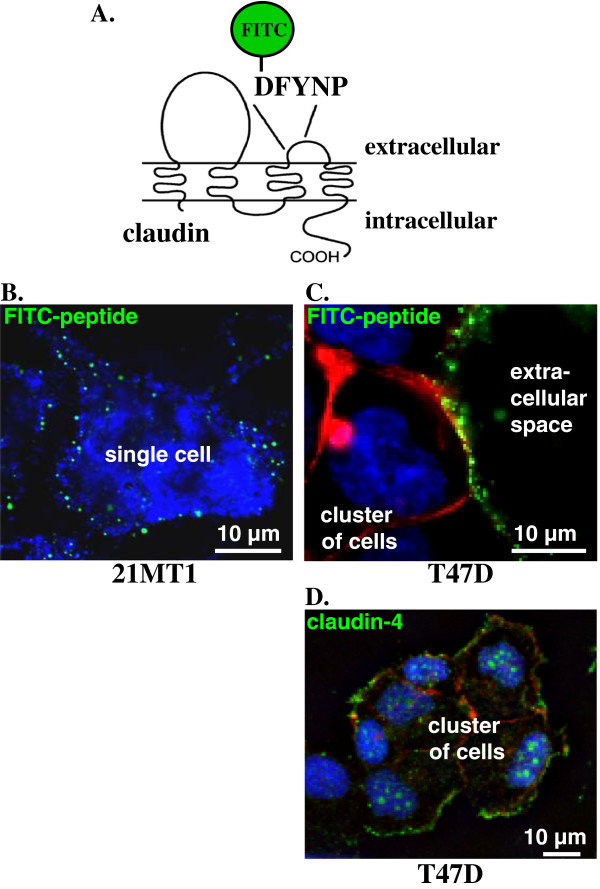
**Claudin is exposed to the extracellular environment in non-polarized cells. **(**A**) Model of the proposed structure of claudin proteins. The FITC-linked DFYNP peptide mimics the DFYNP sequence in the second extracellular loop of the claudin subtypes that express this conserved sequence. 21MT1 (**B**) and T47D (**C**) cells were treated with 400µM FITC-DFYNP for 1 hour at 4°C, washed 3 times with ice-cold PBS and immediately imaged live with a confocal microscope to prevent cellular uptake. Representative images show FITC-DFYNP peptide (green) bound at the surface of 21MT1 and T47D cells when treated and washed at 4°C. Cells were stained with DAPI (**B**&**C**: blue, with leakage of DAPI stain into cytosol of 21MT1 cells) and/or phalloidin (**B**: red). (**D**) FITC-peptide treated T47D cells, at 4°C, were then fixed and treated with a fluorescent antibody directed to claudin-4 (green) and stained with DAPI (blue) and phalloidin (red). Fluorescence was imaged with a confocal microscope.

Cell projections, such as lamelipodia and filopodia, play an important role in cell motility in both normal and tumor cells. Finding claudins in the thread-like projections between normal mammary epithelial cells as well as at the end of filopodia in tumor cells suggested that claudins could potentially be playing a role in directing cell motility. Although claudin-4 puncta are found within and at the end of cellular projections, immunofluorescence staining of fixed cells did not reveal a strong presence of claudin-4 at the plasma membrane. To determine whether claudin-4 can reach the cell surface, at least temporarily, we treated cells with a fluorescently labeled DFYNP mimic peptide (Figure 2A). We have previously characterized the specificity of this small mimic peptide, showing that the fluorescent DFYNP peptide binds to claudin-4-expressing normal mouse (EpH4) mammary epithelial cells at the cell surface at sites of tight junctions [28]. We have also shown that the peptide does not bind to the cell surface of cells that do not express claudins or when glycine is substituted for the any of the last four amino acids of the peptide [28].

Because we have shown that the DFYNP peptide can target claudin-4 we treated tumor cells with the fluorescent mimic peptide at 4°C for one hour before washing the cells with ice cold PBS and subjecting them to immediate imaging. At 4°C, endocytosis is inhibited. Therefore, if claudins are in the membrane at the time of chilling and the extracellular loop is exposed to the extracellular environment, the fluorescent peptide should bind and stay at the membrane to be visualized with the confocal microscope. Fluorescent images show that the peptide does indeed bind to the membranes of both 21MT1 and T47 breast tumor cells (Figure 2B). Because T47D breast cancer cells are known to express high levels of claudin-4, we expected more peptide to be bound to their surface (Figure 2C) compared to the 21MT1 cells. As shown in Figure 2C, our expectations were realized. Although the FITC-peptide is unable to remain attached to cells with fixation and the extensive washings involved in immunofluorescence, we did see a strong fluorescent signal for claudin-4 at the plasma membrane of T47D cells (Figure 2D). These observations suggest that claudins are able to reach the surface of both normal and tumor cells even when they are not associated with traditional tight junction structures. It is important to note that these images are projected images from z-stacks. Due to the thickness of the z-stack for the 21MT1 cells, some apparent internal peptide is, in fact, peptide bound to the upper surface of the cell.

### The second extracellular loop of claudin-4 plays a role in motility

**Figure 3:**
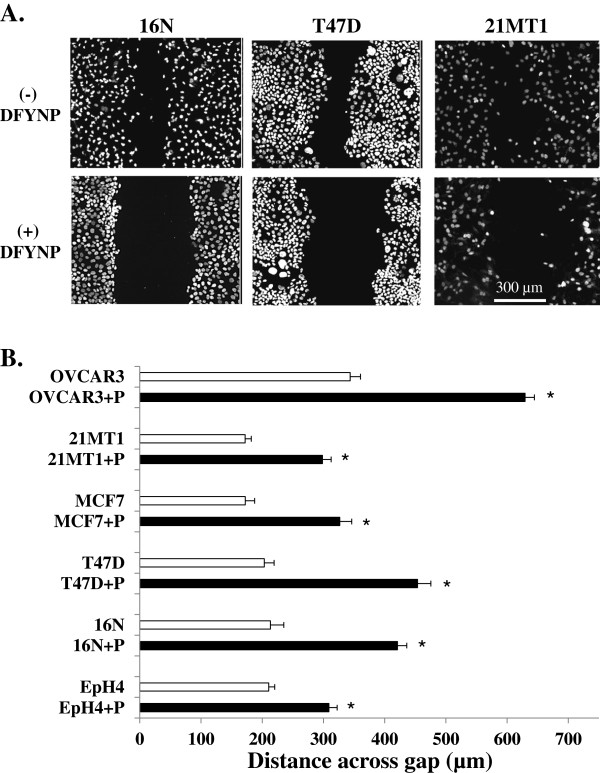
**Disturbing claudin extracellular loop interactions with DFYNP inhibits wound healing.** Representative confocal microscopy images of DAPI (nuclei) staining in 16N, T47D, 21MT1, cells, grown on 100 µg/ml collagen type I + 3.5 µg/cm2 Cell-Tak-coated slides (**A**) at 4 (16N) or 24 (T47D, 21MT1) hours post scratch treatment with control medium (top panel of **A**) or medium with 400µM DFYNP peptide (bottom panel of **A**). The mean distance across the gap at 4 (16N, EpH4) or 24 (T47D, 21MT1, MCF-7, OVCAR3) hours post scratch was measured, using SlideBook software, for each cell line for each condition. Mean ± s.e.m., n=3 wells for each treatment (20 measurements taken for each well) *p < 0.05 vs. non-treated.

To test whether claudins, particularly claudin-4, could play a functional role in directing cell motility through extracellular interactions, we treated normal and tumor cells with the mimic peptide and examined changes in cell motility. We examined motility of normal mammary epithelial cells, using both mouse EpH4 and human 16 N cell lines. We also examined motility in human cancer cells lines that lack normal tight junctional organization as determined by loss of ZO-1 expression (data not shown) and are either known to overexpress claudin-4 (T47D, MCF-7, OVCAR3) or known to be particularly aggressive tumor cells (21MT1). Monolayers, grown on collagen type I plus Cell-Tak-coated glass chamber slides, were wounded by scratching with a pipette tip and the medium was replaced with medium plus or minus 400 μM mimic peptide. We have previously shown that this concentration of peptide disrupts tight junction claudin without altering overall epithelial integrity [28]. Because normal cells show much more rapid wound healing than cancer cells, cells were fixed and imaged after 4 hours post scratch for EpH4 and 16 N cells or 24 hours post scratch for T47D, MCF-7, 21MT1, and OVCAR3 cells. In both normal and cancer cell lines, there was a significant decrease in the rate at which cells moved into the wound when cells were treated with the mimic peptide compared to control cells as shown both in the images in Figure 3A and in the size of the gap quantitated in Figure 3B. These results provide evidence that cell motility is inhibited when claudin extracellular interactions are disturbed in both normal and tumor cell lines, despite the differences in inherent motility.

**Figure 4:**
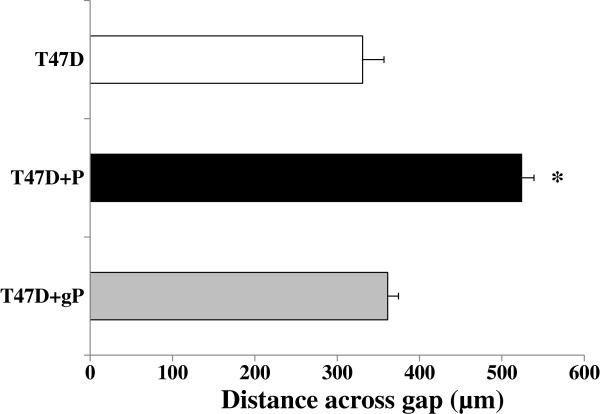
**Peptide with glycine substitution does not inhibit cell motility. **T47D cells were grown to confluence on 100 µg/ml collagen type I + 3.5 µg/cm2 Cell-Tak-coated slides. After the cells were scratched medium was replaced with fresh medium minus peptide (white bar), medium plus 400 µM DFYNP peptide (“+P”, black bar), or medium plus 400 µM DFgNP peptide (“+gP”, gray bar). Mean ± s.e.m., n=4 wells for each treatment (20 measurements taken for each well) *p < 0.05 vs. non-treated.

To test the specificity of the peptide in this effect, we treated wounded T47D cells with a mimic peptide synthesized with glycine substituted for tyrosine (designated “+gP”). The size of the gap remaining after 24 hours was not significantly different between control cells and cells treated with the glycine-substituted peptide (Figure 4). As in Figure 3B, the un-substituted peptide significantly increased the size of the gap.

**Figure 5:**
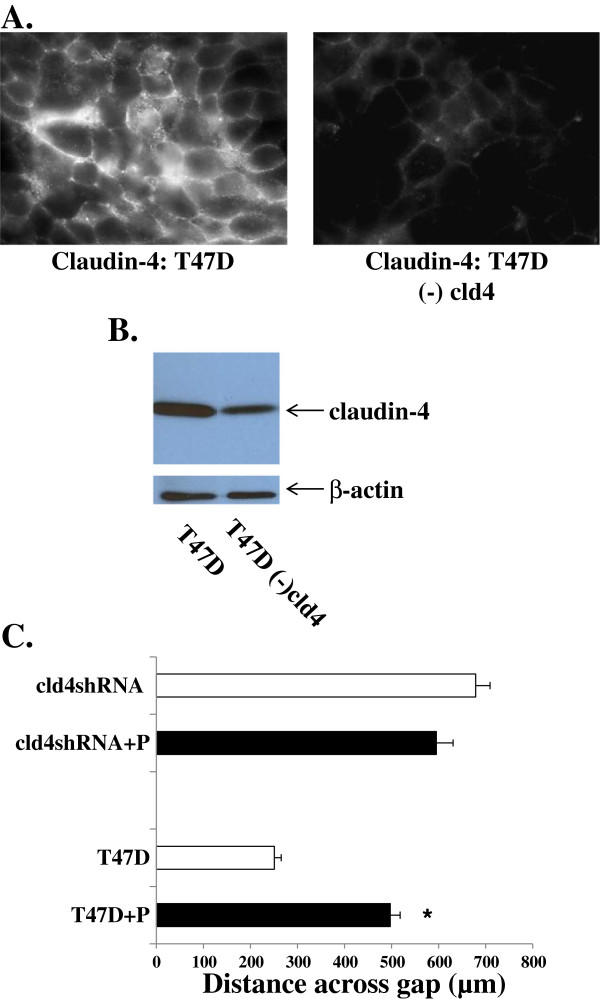
**Claudin-4 knockdown inhibits cell motility. **Representative confocal microscopy images (**A**) of fixed T47D cells and T47D cells with claudin-4 knocked down by shRNA technology [“T47D(-)cld4”] analyzed by immunofluorescence for claudin-4. Western blot analysis (**B**) confirmed a significant decrease in claudin-4 protein expression in T47D(-)cld4 cells compared to T47D cells. A scratch assay was performed on monolayers of both T47D and T47D(-)cld4 in the absence or presence (+P) of 400 µM DFYNP peptide for 24 hours and the distance across the gap at 24 hours was measured (**C**). Mean ± s.e.m., n=4 wells for each treatment (20 measurements taken for each well) *p < 0.05 vs. non-peptide treated.

To confirm that claudin-4 is indeed the target of the peptide that is influencing cell motility, we knocked-down claudin-4 in T47D cells using shRNA technology. Immunofluorescent analysis for claudin-4 in the shRNA treated cells (designated “T47D(-)cld4”) showed very little claudin-4 protein; only isolated cell patches of cells displayed low-levels of claudin-4 (Figure 5A). Western blot analysis (Figure 5B) confirmed a significant decrease in claudin-4 expression in the T47D(-)cld4 cells compared to T47D cells. Scratch assays were then performed on T47D versus T47D(-)cld4 in the presence or absence of 400 μM mimic peptide. In the absence of the mimic peptide, T47D(-)cld4 cells moved more slowly to heal the wound than T47D cells (Figure 5C, open bars), confirming that loss of claudin-4 inhibits cell motility. Treatment of T47D(-)cld4 cells with the mimic peptide had no effect on cell motility, confirming that claudin-4 is the target of the mimic peptide that is influencing cell motility.

### Extracellular matrix proteins important for claudin-facilitated cell motility

**Figure 6:**
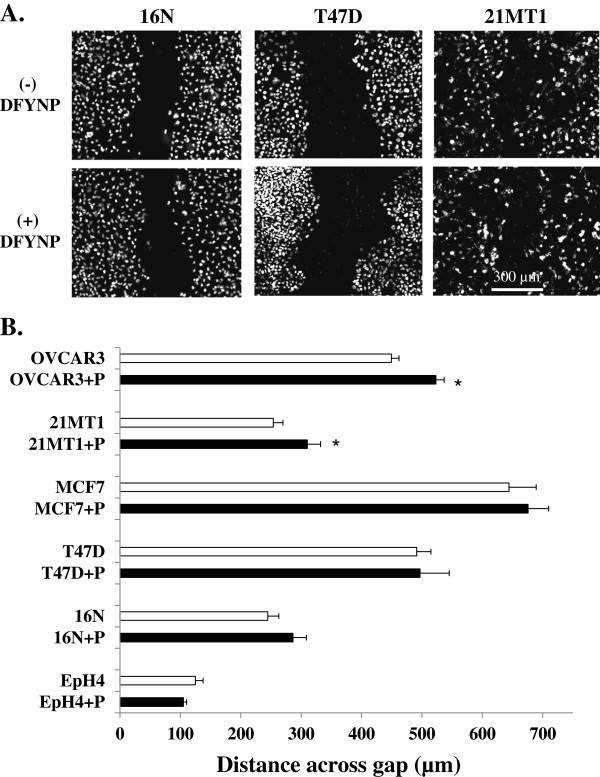
**Disruption of wound healing by the peptide is seen only in the presence of collagen.** Representative confocal microscopy images of DAPI (nuclei) staining of normal 16N human mammary epithelial cells and T47D and 21MT1 breast tumor cells, grown on 3.5 µg/cm2 Cell-Tak-coated slides (**A**) at 4-24 hours post scratch treatment with control medium (top panel of **A**) or media with the 400 µM DFYNP claudin mimic peptide (bottom panel of **A**). The size of the gap at 4 (normal cells: 16N, EpH4) or 24 (tumor cells: T47D, 21MT1, MCF-7, OVCAR3) hours post scratch was measured, using SlideBook software, for each cell line for each condition. Mean ± s.e.m., n=3 wells for each treatment (20 measurements taken for each well) *p < 0.05 vs. non-treated.

To determine whether claudins interact with or require specific extracellular matrix proteins to promote motility, normal and tumor cells were grown on glass chamber slides coated with Cell-Tak, a non-physiological matrix derived from mollusk polyphenolic proteins. Interestingly, when grown on Cell-Tak, the EPH4 cells migrated much more quickly into the scratch and the 16 N cells moved at about the same speed (compare the length of the corresponding open bars in Figures 3 and 6). However, the mimic peptide did not inhibit this movement (Figure 6A and B), with a similar sized gap for treated and untreated cells. The tumor cells moved much more slowly on Cell-Tak compared to collagen (again compare the lengths of the open bars in Figures 3 and 6), with only 21MT1 and OVCAR3 cells showing a small, but significant, difference in gap size between treated and untreated cells. These data suggest that although normal and tumor cells behave differently on different extracellular matrices, the motility of both cell types involves an extracellular interaction of claudin that appears to require collagen matrix.

**Figure 7:**
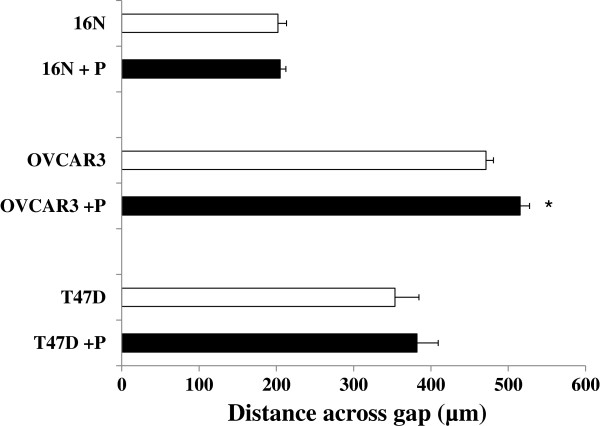
**Wound healing response to peptide is specific for collagen. **Normal human mammary epithelial (16N) as well as breast (T47D) and ovarian (OVCAR3) tumor cell monolayers, grown on 30 µg/ml fibronectin-coated glass chamber slides, were scratched and the size of the gap remaining at 4 (16N) or 24 (T47D, OVCAR3) hours was measured using SlideBook software. The size of the gap was compared between non-treated cells (white bars) and cells treated with 400 µM mimic peptide. Mean ± s.e.m., n=4 wells for each treatment (20 measurements taken for each well) *p < 0.05 vs. non-treated.

To further investigate the potential requirement for specific extracellular matrices in claudin-facilitated motility, we examined movement of select cells on fibronectin. Normal human mammary epithelial cells (16 N), breast tumor cells (T47D) and ovarian tumor cells (OVCAR3) were all grown on fibronectin-coated glass slides. Confluent monolayers were scratched and the size of the gap remaining at 4 hours (16 N) or 24 hours (T47D, OVCAR3) was measured. T47D cells moved more rapidly on fibronectin than on Cell-Tak. However, OVCAR3 and 16 N cells moved at about the same speed judging from the size of the gap on the two matrices. However, motility of all three cell lines was unchanged in the presence or absence of the claudin mimic peptide (Figure 7).

### Proliferation and apoptosis are not playing a significant role in the inhibition of motility

**Figure 8:**
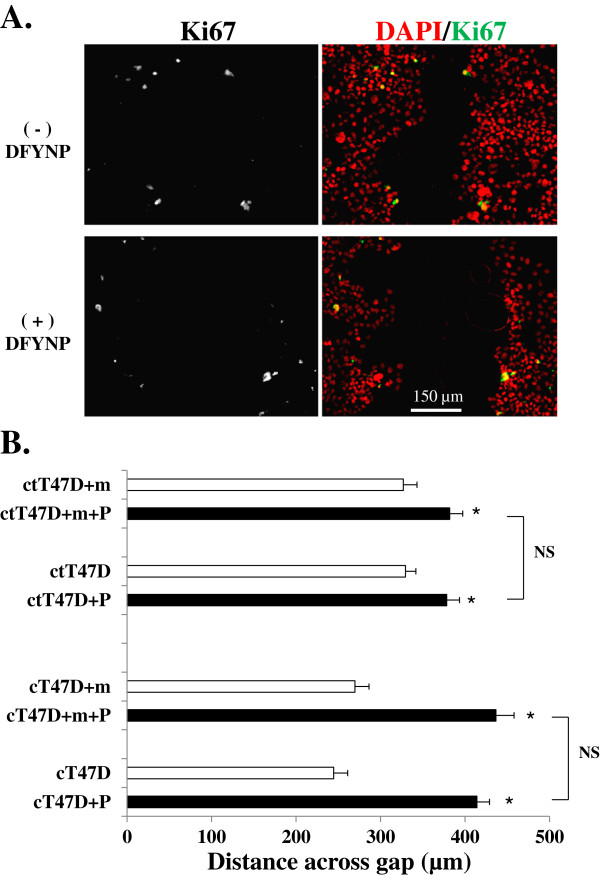
**Proliferation does not influence the wound healing response. **Representative confocal microscopy images of nuclei (DAPI, red) and Ki67 (yellow) in T47D cells, grown on 100 µg/ml collagen type I + 3.5 µg/cm2 Cell-Tak, 24 hours post scratch in absence or presence of the mimic peptide (**A**). The size of the gap remaining at 24 hours post scratch treatment was measured for T47D cells plated on Cell-Tak (ctT47D) or collagen (cT47D). After the scratch the media was replaced with either normal media plus (+P) or minus DFYNP peptide in the absence or presence (+m) of mitomycin-C. The inhibition of wound healing with the DFYNP peptide was similar in cells treated with mitomycin-C compared to untreated cells. Mean ± s.e.m., n=3 wells for each treatment (20 measurements taken for each well), *p < 0.05 vs. non-treated, NS=not significant.

To confirm that inhibition of proliferation is not playing a role in the reduced wound closure response induced by the mimic peptide, T47D cells were treated with an antibody directed to Ki67, a marker of cell proliferation. A similar number of Ki67 positive cells was seen in treated cells compared to untreated cells (Figure 8A), suggesting a similar rate of proliferation in both conditions. In a separate set of experiments, cells were treated with mitomycin-C for two hours prior to wounding as well as during treatment with the mimic peptide to inhibit cell proliferation. In the presence of mitomycin-C, the effects of the mimic peptide were similar to those seen in the absence of mitomycin-C (Figure 8B). When cells were grown on Cell-Tak, only a small effect of the peptide was seen, consistent with the results in Figure 6B, whereas a highly significant effect of peptide was seen on the collagen matrix as before. Importantly mitomycin-C had no significant effect in the size of the gap in peptide treated cells on either matrix. These results indicate that cell proliferation is not an important part of the response to wounding under any conditions, since mitomycin C treatment did not alter the size of the gap under any of the observed conditions.

To determine whether increased apoptosis could be playing a role in the reduced rate of wound repair seen with the peptide, caspase-3 activation was examined in both untreated and DFYNP treated cells. Immunofluorescence of active caspase-3 revealed a low and insignificantly different level of caspase-3 activation in untreated (2.34 ± 0.43% of the total cell population stained positive for caspase-3 activation) and treated (3.27 ± 0.38% of the cell population stained positive for caspase-3 activation) T47D cells grown on collagen plus Cell-Tak. Importantly, caspase-3 activation was seen throughout the monolayer and was not restricted to the edges of the scratch (data not shown); further indicating that increased apoptosis is not playing a role in the peptide-induced change in cell motility.

## Discussion

In this study we have shown that claudin-4 can be found in puncta along cellular projections of non-confluent normal mammary epithelial cells as well as breast tumor cells. Claudin-4 can reach the surface of these cells, where the second extracellular loop is exposed to the extracellular environment in the absence of established tight junction structures. Both disturbing normal second extracellular loop interactions with a mimic peptide and knock-down of claudin-4 expression inhibited wound healing, providing strong evidence for a role for claudin-4 in cell motility when cells are grown on extracellular matrices containing collagen.

These results are consistent with previous reports in which claudin expression was associated with changes in cell motility [15-19]. Agarwal et al. [15] showed that forced expression of claudin-4 in cells that don’t normally express claudin-4 increased cell motility. We have shown that claudin-4 can also promote motility in cells that normally express claudin-4 and that both normal cells and different types of tumor cells all exhibit the same phenomenon. It is, therefore, possible that claudin-4-faciltated motility may represent a fairly common mechanism used by cells of epithelial origin to direct cell movement.

The motility slowing effect of the function-blocking peptide, DFPNY, provides the first direct evidence that claudin-4 is promoting cell motility through interactions of its extracellular loop. The finding that the effect was only seen, in our experiments, when collagen was included in the extracellular matrix provides strong evidence to support the notion that the interaction includes matrix proteins. The nature of this interaction will require more detailed investigation. It is possible that claudin-4 interacts directly with collagen to promote cell motility or it may interact with other proteins that are known to interact with collagen. Ding et al. [5] showed that claudin-1 and claudin-7 form a complex with integrin α2 at the basolateral membrane of normal mouse intestine. Integrins are well known receptors for extracellular matrix proteins, with integrin α2 a known receptor for collagen. Additionally, claudin-7 has been shown to complex with a CD44 in colorectal cancer cells [19,30]. CD44 is a surface glycoprotein that has been shown to bind to hyaluronan, as well as collagen, to promote tumor cell motility [31]. Although a direct interaction of claudin-4 with CD44 has yet to be confirmed, CD44 expression has been found to be associated with claudin-4 expression in particularly aggressive ovarian tumor cells [32]. Claudin expression has also been associated with matrix metalloproteinases (MMPs). MMPs are secreted zinc-endopeptidases that are known to degrade extracellular matrix and enhance cell motility, particularly in collective cell migration [33]. An investigation of HOSE cells transfected with claudin-3 or -4 showed an increase in MMP-2 activity in cells expressing the claudins versus normal non-expressing HOSE cells [15]. Takehara et al. [34] also observed increased MMP-2 and MMP-9 activity in Caco-2 cells over expressing claudin-3 or -4 compared to mock transfected Caco-2 cells. These observations, along with a report by Miyamori et al. [20] that claudins have the potential to interact with MT1-MMP to activate MMP-2, suggest that claudins may increase MMP activity to digest extracellular matrix and promote cell motility. All these observations are consistent with a critical role of collagen for claudin-enhanced motility.

The focus of this study has been on claudin-4. It is possible that other claudin subtypes may be involved in cell motility, especially those subtypes with the DFYNP sequence. Claudin-3 has been shown to increase cell motility when expressed in ovarian surface epithelial cells that don’t normally express claudin-3 [15]. Interestingly, claudin-5 and claudin-11 have been implicated in promoting motility, but do not have the DFYNP sequence in the second extracellular loop. It is possible that these claudin subtypes use a different mechanism to promote motility and may even take advantage of other key domains of claudin structure such as the cytosolic PDZ-binding domain. It is even possible that the extracellular loop of claudin-4 may promote motility through initiation of intracellular signaling pathways.

Although it is still unclear how claudins may promote cell motility, it is becoming clear that claudins are indeed playing an important role in promoting cell movement during wound healing. Why claudins play a role in cell motility is another unresolved question. However, our hypothesis is that non-junctional claudins have lost their normal connections to a partner claudin through the lack of a neighboring cell. They may, therefore, be sampling the extracellular environment and directing cell movement to find a partner. This process would be critical in wound repair when epithelial cells are moving in to quickly cover defenseless tissue and re-establish the protective barrier that epithelial cells provide. It is interesting to note that an examination of gene expression during normal mammary gland development showed a sharp increase in claudin-3 and -4 expression during early days of involution [35,36], a time of significant tissue remodeling. Could these claudins have a designated role in cell motility during this window when both dissolution and healing of the mammary epithelium is taking place? Is it possible that this process becomes significantly disrupted in tumor cells? Perhaps these non-junctional claudins in tumor cells are unable to interact with neighboring claudins, leading them to continue to migrate in undesired directions. Clearly, many questions remain about how claudins promote cell motility, especially since they are traditionally thought of as stabilizing proteins. However, the relevance to normal wound healing responses as well as tumor progression opens an exciting new area in the understanding of claudin function.

## Conclusions

In summary, we have shown that claudin-4 promotes both normal and tumor cell motility through key extracellular loop interactions. Claudin-4 can be found in cellular projections, where it can cycle from the cytosol to the membrane and expose the DFYNP sequence of the second extracellular loop to the extracellular environment. In the absence of a neighboring cell, the second extracellular loop then interacts either directly or indirectly with collagen to direct cell movement.

## Methods

### Cell culture

Primary mammary epithelial cells were isolated from the mammary glands of female FVB mice (Jackson Laboratories, Bar Harbor, Maine, USA). The fourth and fifth mammary glands were dissected from pregnancy day 15 dams. The minced glands were placed in collagenase solution consisting of: Dulbecco’s Modified Eagle Medium: Nutrient Mixture F-12 (DMEM/F12) media (Mediatech, Manassas, VA), 2 mg/ml Collagenase A (Roche Applied Science, Indianapolis, IN), and 50 μg/ml Gentamycin (Mediatech), and incubated at 37°C with shaking at 200 rpm for 2 hours. Cells were then spun at 1500 rpm for 10 minutes and the pellet was pulse washed with PBS (with calcium and magnesium) 5 to 10 times at 1500 rpm for 2 seconds. The pellet, which contains mammary epithelial organoids, was then resuspended in growth media containing: DMEM/F12 media, 1X ITS (10X stock, Sigma, St. Louis, MO), 100 μg/ml EGF (BD Biosciences, San Diego, CA), 5% FBS (Mediatech), 50 μg/ml gentamycin (Mediatech), 1% penicillin/streptomycin (Mediatech), and 2.5 μg/ml FUNGIZONE (Gibco, Grand Island, NY); and plated on 8-well chamber slides coated with Collagen I (Sigma, St. Louis, MO).

EpH4 cells were grown in Dulbecco’s Modified Eagle’s Medium (DMEM) supplemented with 5% heat-inactivated fetal bovine serum (FBS), 1% penicillin/streptomycin, and 10 mM Hepes (Mediatech). 16 N, 21PT and 21MT1(kindly provided by Heide L. Ford) cells were grown in DMEM supplemented with 10% heat-inactivated FBS, 1% penicillin/streptomycin, 1% non-essential amino acids (Mediatech), 1 mM sodium pyruvate (Mediatech), 2 mM L-glutamine (Mediatech), 10 mM Hepes, 1 μg/ml insulin (Sigma), 1 μg/ml hydrocortisone (Sigma), and 12.5 ng/ml EGF. T47D cells were grown in RPMI (Mediatech) media supplemented with 10% FBS, and 6 ng/ml insulin. MCF7 cells were grown in Minimum Essential Media (MEM, Mediatech) supplemented with 10% heat-inactivated FBS, 1% penicillin/streptomycin, 1% non-essential amino acids (Mediatech), 1 mM sodium pyruvate (Mediatech), 2 mM L-glutamine (Mediatech), and 10 μg/ml insulin. OVCAR3 cells (kindly provided by Monique A. Spillman) were grown in DMEM supplemented with 10% FBS. Cells were trypsinized (0.25% Trypsin, EDTA, Mediatech) and plated every 3–4 days. Cells were plated, 2×10^4^ cells/well (21PT, 21MT1, T47D, MCF7, OVCAR3) or 1×10^4^ cells/well (EpH4, 16 N) onto Lab-Tek glass 8-chamber slides (NUNC, Rochester, NY) for experiments. Chamber slides were coated with 150 μl of either Collagen type I (100 μg/ml, Sigma), fibronectin (30 μg/ml, Sigma), Cell-Tak (1:100 dilution in PBS, BD BioSciences, Bedford, MA), or Collagen I plus Cell-Tak (1:100 dilution of Cell-Tak in Collagen I). Slides were then incubated at 37°C for 30 min (Collagen type I and/or Cell-Tak) or RT for 45 min (fibronectin) before washing with sterile water and drying under UV for 20 min.

### Immunofluorescence

Cell monolayers were fixed with 2% paraformaldehyde for 15 min at room temperature. Cells were then permeabilized with 0.5% Triton X-100 for 5 minutes before blocking with 2% BSA for one hour. Cells were treated with rabbit anti-claudin-4 (1:200, Zymed, Carlsbad CA), rat anti-ZO-1 (1:50, Santa Cruz Biotechnology), and/or rabbit anti-Ki67 (1:100, Abcam, Cambridge, MA) primary antibodies for 1 hour. After washing with Phosphate Buffered Saline (PBS), cells were treated with donkey anti-rabbit-CY3, donkey anti-rat-FITC (1:150, Jackson ImmunoResearch Laboratories, West Grove, PA), Alexa Fluor® 647 Phalloidin (7.5 units/ml, Invitrogen, Carlsbad, CA), and/or DAPI (5 μg/ml, MP Biochemicals, Solon, OH) for 45 minutes. Monolayers were then washed five times, five minutes each, with PBS and OPDA (20 mg/ml, o-phenylenediamine dihydrochloride in 1 M Tris, pH 8.5) was applied before addition of a coverslip. Fluorescence was imaged on an Olympus Spinning Disk confocal microscope, using SlideBook software (Intelligent Imaging Innovations, Inc., Denver, CO, USA).

### FITC-linked peptide

A FITC labeled DFYNP peptide (FITC-DFYNP-amide, D-amino acid form) was synthesized in the Peptide and Protein Chemistry Core, University of Colorado Anschutz Medical Campus as described previously [28]. Cells were treated with 400 μM FITC-DFYNP peptide for 1 hour on ice before washing three times with ice-cold PBS and immediately imaging. Fluorescence was imaged on an Olympus Spinning Disk confocal microscope, using SlideBook software (Intelligent Imaging Innovations, Inc., Denver, CO, USA).

### Scratch assay

Cells were grown on collagen I, Cell-Tak, or collagen plus Cell-Tak until cells reached confluence (usually 5 days in culture). A 200 μM pipette tip was used to scratch the monolayers, creating a vertical cell-free gap. Media was immediately changed to normal growth media or growth media plus 400 μM DFYNP mimic peptide or DFGNP mutated peptide. Cells were incubated at 37°C for 4 hours (EpH4, 16 N) or 24 hours (21PT, 21MT1, T47D, MCF7, OVCAR3) before fixing with 2% paraformaldehyde for 15 min at room temperature. Cells were treated with Alexa Fluor® 647 Phalloidin and DAPI, washed with PBS, coverslipped, and imaged as described above. Micrographs of two fields of interest, near the center of the slide, were taken and SlideBook software was used to measure the thickness of the gap (20 measurements along the length of each imaged scratch were taken for each well under each condition).

### shRNA knockdown

T47D breast tumor cells were plated at 3.2 × 10^4^ in a 96-well plate and incubated at 37°C for 24 hours. When cells were 70% confluent, 15 μl of claudin-4 shRNA lentiviral suspension (University of Colorado Functional Genomics Facility, Boulder, CO, USA) was added to each well and cells were incubated overnight at 37°C. Medium was then removed and replaced with fresh medium. Twenty-four hours later the cells were treated with 2 μg/ml puromycin to select for transduced cells. Colonies of cells were selected and expanded for experiments. Western blot analysis was performed to select cells with most significant reduction in claudin-4 expression. Knockdown of claudin-4 expression was confirmed with immunofluorescence analysis of the cultured cells as described above. This experiment also confirms the specificity of the antibody.

### Statistics

Data are presented as Mean ± Standard Error of the Mean (s.e.m.). An unpaired Student *t* test was used for statistical comparison between control and treatment groups. A *p* value of < 0.05 was considered significant.

## Abbreviations

DFYNP: Aspartic acid-phenylalanine-tyrosine-asparagine-proline; FITC: Fluorescein isothiocyanate; MMP: Matrix metalloproteinase; DMEM/F12: Dulbecco’s modified eagle medium, nutrient mixture F-12; RPMI: Rosewell park memorial institute medium; FBS: Fetal bovine serum; EGF: Epidermal growth factor; RT: Room temperature; BSA: Bovine serum albumin; PBS: Phosphate buffered saline; OPDA: O-phenylenediamine dihydrochloride.

## Competing interests

The authors declare no competing interests.

## Authors’ contributions

HKB conceived the hypothesis and experimental design. HKB and PGW performed the experiments. Analysis and interpretation of data was done by HKB and MAS. HKB wrote the manuscript, with critical edits by MAS. All authors read and approved the final manuscript.
